# Continuous Infusions of Meropenem in Ambulatory Care: Clinical Efficacy, Safety and Stability

**DOI:** 10.1371/journal.pone.0102023

**Published:** 2014-07-14

**Authors:** Laurens Manning, Cameron Wright, Paul R. Ingram, Timothy J. Whitmore, Christopher H. Heath, Ingrid Manson, Madhu Page-Sharp, Sam Salman, John Dyer, Timothy M. E. Davis

**Affiliations:** 1 School of Medicine and Pharmacology, University of Western Australia, Fremantle Hospital, Fremantle, Western Australia, Australia; 2 Department of Infectious Diseases, Fremantle Hospital and Health Service, Fremantle, Western Australia, Australia; 3 Pharmacy Department, Fremantle Hospital and Health Service, Fremantle, Western Australia, Australia; 4 Department of Microbiology and Infectious Diseases, Royal Perth Hospital, Perth, Western Australia, Australia; 5 School of Pathology and Laboratory Medicine, University of Western Australia, Perth, Western Australia, Australia; 6 School of Medicine and Pharmacology, University of Western Australia, Royal Perth Hospital, Perth, Western Australia, Australia; 7 School of Pharmacy, Curtin University, Bentley, Western Australia, Australia; Fondazione IRCCS Ca' Granda Ospedale Maggiore Policlinico, Università degli Studi di Milano, Italy

## Abstract

**Objectives:**

Concerns regarding the clinical impact of meropenem instability in continuous infusion (CI) devices may contribute to inconsistent uptake of this method of administration across outpatient parenteral antimicrobial therapy (OPAT) services.

**Methods:**

We retrospectively reviewed the clinical efficacy and safety of CIs of meropenem in two Australian tertiary hospitals and assessed its stability under simulated OPAT conditions including in elastomeric infusion devices containing 1% (2.4 g) or 2% (4.8 g) concentrations at either ‘room temperature’ or ‘cooled’ conditions. Infusate aliquots were assayed at different time-points over 24 hours.

**Results:**

Forty-one (82%) of 50 patients had clinical improvement or were cured. Adverse patient outcomes including hemato-, hepato- and nephrotoxicity were infrequent. Cooled infusers with 1% meropenem had a mean 24-hour recovery of 90.3%. Recoveries of 1% and 2% meropenem at room temperature and 2% under cooled conditions were 88%, 83% and 87%, respectively. Patients receiving 1% meropenem are likely to receive >95% of the maximum deliverable dose (MDD) over a 24-hour period whilst patients receiving 2% meropenem should receive 93% and 87% of the MDD under cooled and room temperature conditions, respectively.

**Conclusions:**

Meropenem infusers are likely to deliver ∼95% MDD and maintain effective plasma concentrations throughout the dosing period. These data reflect our local favourable clinical experience with meropenem CIs.

## Introduction

Administration of antibiotics by continuous infusions (CI) is a practical method in outpatient parenteral antimicrobial therapy (OPAT) settings. Ideally, drug stability should be maintained in the CI device throughout the infusion period to ensure that the patient receives sufficient active drug to achieve cure while avoiding exposure to toxic degradation products. Although the European and US Pharmacopoeias define infusion stability as the maintenance of >90% of the initial concentration throughout the infusion period, this may not necessarily apply for certain medications and might limit therapeutic opportunities for these drugs [1].

One such drug might be meropenem, a broad spectrum carbapenem antibiotic that is increasingly required for the treatment of severe infections caused by multi-resistant Gram-negative bacteria [2]. Meropenem stability in solution depends on infusion time, concentration and temperature. Significant degradation is observed at higher ambient temperatures, when diluted in normal saline and stored over 8 hours [3], in sealed vials over 24 hours at 25°C and 37°C, and in infusion devices at higher concentrations (6.4%) stored at 37°C [3]–[6]. However, these studies have also demonstrated improved stability at lower concentrations (<4%) and ambient temperatures (<25°C) [4], [7]–[9].

Uncertainty regarding the clinical impact of meropenem stability is reflected by different practices across OPAT services internationally [10]. In Western Australian tertiary hospitals, we have accumulated clinical experience using 24-hour CI of meropenem in our OPAT services without evident safety or efficacy concerns. In light of data demonstrating a lack of stability of meropenem and variability in clinical CI use, we performed a study with two components, namely a retrospective review of the efficacy and safety of CI meropenem in our OPAT services, and meropenem stability under simulated real-world conditions using the infusion devices used by our OPAT service and at lower concentrations than in previous studies.

## Materials and Methods

### Efficacy and safety review

The study setting was two large adult tertiary hospitals located in a temperate region of Western Australia, where the annual mean maximum and minimum temperatures are 24.7°C and 12.7°C, respectively [11]. All adult patients who received at least one day of meropenem via 24-hour CI in our respective OPATs between July 2008 and March 2013 were eligible for inclusion. Patients typically received meropenem via intermittent dosing as an inpatient before being switched to an outpatient 24-hour CI at the same daily dose. Meropenem was delivered via a peripherally inserted central catheter (PICC) using an elastomeric infuser device prepared locally (LV10, Baxter Healthcare, Sydney, Australia). The prescribed meropenem dose was reconstituted in 240 mL of 0.9% normal saline and delivered at 10 mL/h. Active cooling of the infuser devices was not routinely performed. Patients were reviewed by nurses daily, and had weekly physician review together with weekly monitoring of hematologic, renal and hepatic function. Case records of patients were reviewed to determine demographics, co-morbidities, indication for antimicrobial therapy, duration and dose of meropenem, concomitant antimicrobials, laboratory results and adverse events. Clinical outcome was assessed at the completion of parenteral therapy and categorised as follows: cure (completed OPAT therapy with resolution of infection and no requirement for long term antibiotic therapy), improved (complete OPAT therapy with partial resolution of infection but need for further follow-up) or failure (progression or non-response of infection despite OPAT, re-admission, surgical intervention, or all cause death). We defined antibiotic-induced neutropaenia, eosinophilia, thrombocytopaenia and anaemia as the development of an absolute neutrophil count <1×10^9^/L, eosinophil count >0.5×10^9^/L, platelet count <150×10^9^/L and haemoglobin <100 g/L, respectively. Nephrotoxicity was defined as a rise in creatinine of >50% from baseline [12]. Hepatotoxicity was defined using US FDA criteria [13]. Approval from the South Metropolitan Area Human Research Ethics Committees was obtained for both study components. Patients recruited to the stability study provided written, informed consent. For the retrospective arm of the study, individual consent was not obtained, but all records were anonymised and de-identified prior to analysis.

### Meropenem stability under simulated OPAT conditions

Meropenem trihydrate (Ranbaxy Australia Pty Ltd, North Ryde, Australia) was reconstituted in normal saline and placed in an elastomeric infuser device (LV10, Baxter Healthcare, Sydney, Australia) at either 1% (2.4 g in 240 mL) or 2% (4.8 g in 240 mL) w/v. These concentrations were chosen to be comparable to other stability studies (using rounded up by % w/v) as well as being within the median dose ranges that were given to the majority of our patients. Infusers were refrigerated overnight and then allocated to ‘room temperature’ or ‘cooled’ by placing them in a standard carry-bag or a ‘cooler bag’ (Fridge-to-go, Interaction Branding Pty Ltd, Frenchs Forest, Australia) with a puck-shaped ice brick. Continuous temperature monitoring strips (TP138, timestrip PLUS, Timestrip UK Ltd, London, UK) were placed on the outside of each infuser as a semi-quantitative measure of the time that each infuser spent above 20°C.

Volunteers carried the infusers around the waist or over the shoulder for a 24-hour period to simulate real-world OPAT conditions. This allowed ambient temperature to vary throughout the day. Volunteers were also instructed to place the infusion devices outside the bedclothes whilst sleeping. For ‘cooled’ infusers, the ice pucks were changed every 8 h.

Infusates were sampled at 0, 2, 4, 8, 14 and 24 hours by allowing a small amount (∼2 mL) to flow from the device before collecting 1.5 mL for testing. Infusate aliquots were transported on ice and stored at −80°C prior to analysis.

Meropenem (molecular weight [MW]  = 437.51) was assayed by liquid chromatography-mass spectroscopy (LC/MS) with ertapenem sodium (MW = 497.51) (Merck & Co. Inc, Rahway, USA) as an internal standard (IS). Chromatographic separation was performed on a GraceSmart RP 18 3 µm column (100 mm×2.1 mm i.d.) at ambient temperature. The autosampler temperature was set at 4°C to protect sample degradation during analysis. The retention times (RT) for meropenem and ertapenem were 1.8 and 2.5 min, respectively. A 5-point linear calibration curve (r^2^≥0.99) was constructed by spiking meropenem into blank 0.9% w/v saline solution in a range of 0.01–0.05% w/v.

Stock solutions of meropenem and ertapenem, were prepared separately and stored protected from light at −80°C. A 5-point linear calibration curve (r^2^≥0.99) was constructed by spiking meropenem into blank 0.9% NaCl in a range of 0.01–0.05%. Etrapenem IS (0.02%) was added to all samples. Infusor samples were diluted 50 times with normal saline. The injection volume was 1 µL and each sample was injected twice. Chromatographic data (peak area ratio of meropenem:ertapenem) were processed using LAB Solution (Version 5, Shimadzu, Japan). Assay intra- and inter-day relative standard deviations were below 10% and the limit of detection was 0.00001%.

Data were analysed using GraphPad Prism (version 5.0a). Continuous variables were summarised as mean±SD or median (interquartile range [IQR]) for parametric and non-parametric data, respectively. Two-sample comparisons of continuous variables were by Student's *t*-test and associations between normally-distributed variables were assessed by Pearson's correlation co-efficient. The observed area under the concentration-time curve (AUC) was calculated using the trapezoidal method. This value was then divided by the theoretical maximum AUC (assuming no degradation of meropenem), giving a percentage of the maximum deliverable dose (MDD).

An illustrative simulation was then performed using previously described meropenem population pharmacokinetic parameters [14]. Stability data from the present study was incorporated into a custom model (using ADVAN 6) where the input rate of meropenem followed the reduction in concentration noted over 24 hours.

Combining this custom input model with the published population pharmacokinetic model, median plasma meropenem concentrations (as well as the 2.5^th^ and 97.5^th^ centiles, to provide a 95% prediction interval [PI_95_]) for 200 simulations of an average 70 kg adult male with normal renal function during a 7-day course of meropenem given by CI were simulated using NONMEM (version 7.2.0) and plotted against microbiological breakpoints for *Pseudomonas aeruginosa* (4 mg/L) and Enterobacteriaceae (1 mg/L) [15].

## Results

### Efficacy and safety review

Fifty patients received CI of meropenem through our OPAT services during the 6-year study period. The median age was 59 (range 18–91) years and 60% were male. Patients had a range of infections including lower respiratory tract (n = 17), bone and/or joint (n = 14), intra-abdominal (n = 6), diabetic foot (n = 4), urinary tract (n = 3), otitis externa (n = 2) and other (n = 4). The most prevalent co-morbidities were diabetes mellitus (n = 18), cardiac disease (n = 13), chronic renal impairment (n = 11) and immuno-suppression (n = 10). The most frequent primary pathogens were *P. aeruginosa* (n = 30) and other Gram-negative organisms (n = 8), and *Nocardia* species (n = 2) and *Burkholderia pseudomallei* (n = 1) were also isolated. Infection was polymicrobial in 40% of cases. Twenty-four patients (48%) received a second antibiotic for additional activity against Gram-negative bacteria. These included fluoroquinolones (n = 12), aminoglycosides (n = 7), folate antagonists (n = 4) and inhaled colistin (n = 1). The median duration of meropenem therapy as an inpatient prior to OPAT was 7 (IQR 2.25–10, range 0–41) days and the median OPAT duration was 18 (IQR 9–29, range 4–81) days. The median total daily dose of meropenem was 3 (IQR 3–3, range 1–6) g or 1.25 (range 0.42–2.5)% w/v. Thirty-six patients had surgical debridement as part of their inpatient management in addition to antibiotic therapy. For most patients (92%), infusers were not actively cooled.

At completion of meropenem therapy, 8 patients (16%) were cured, 33 (66%) had improved and 9 (18%) failed therapy. OPAT CI with meropenem was ceased early in 5 patients (10%). A meropenem-resistant isolate was identified in two of these cases, while the remaining three were readmitted and CI with meropenem interrupted due to dyspnoea, neutropaenia and toxicity due to concomitant voriconazole, respectively.

Asymptomatic thrombocytopaenia and eosinophilia were observed in 2 (4%) and 4 (8%) patients, respectively, but did not necessitate cessation of therapy. Six patients (12%) were anaemic at baseline and a further two (4%) developed anaemia during therapy. Elevations in serum alanine amino transferase (ALT) to above the upper limit of normal (ULN; >40 U/L) occurred in 5 (10%) of patients. However, no patients had criteria under Hy's Law for drug induced liver injury (ALT >3× ULN together with a rise in serum bilirubin to >2× ULN) [13].

Neutropaenia developed in one patient, a 90-year old male with infective endocarditis due to *Enterobacter cloacae*. He had received meropenem by CI for 43 days before developing a nadir neutrophil count of 0.06×10^9^/L. The neutropaenia resolved on cessation of meropenem. Nephrotoxicity developed in one patient, an 86-year old woman with a history of chronic renal impairment and type-2 diabetes. She was receiving concomitant voriconazole for malignant otitis externa and was re-admitted to hospital for side-effects attributed to this antifungal agent. Meropenem, given by intermittent dosing, was continued in hospital with resolution of her renal impairment. This patient was also one that developed anaemia. There were no PICC-related adverse events, infusion reactions or neurotoxicity, and no patients died during OPAT therapy.

### Meropenem stability under simulated OPAT conditions

**Figure 1:**
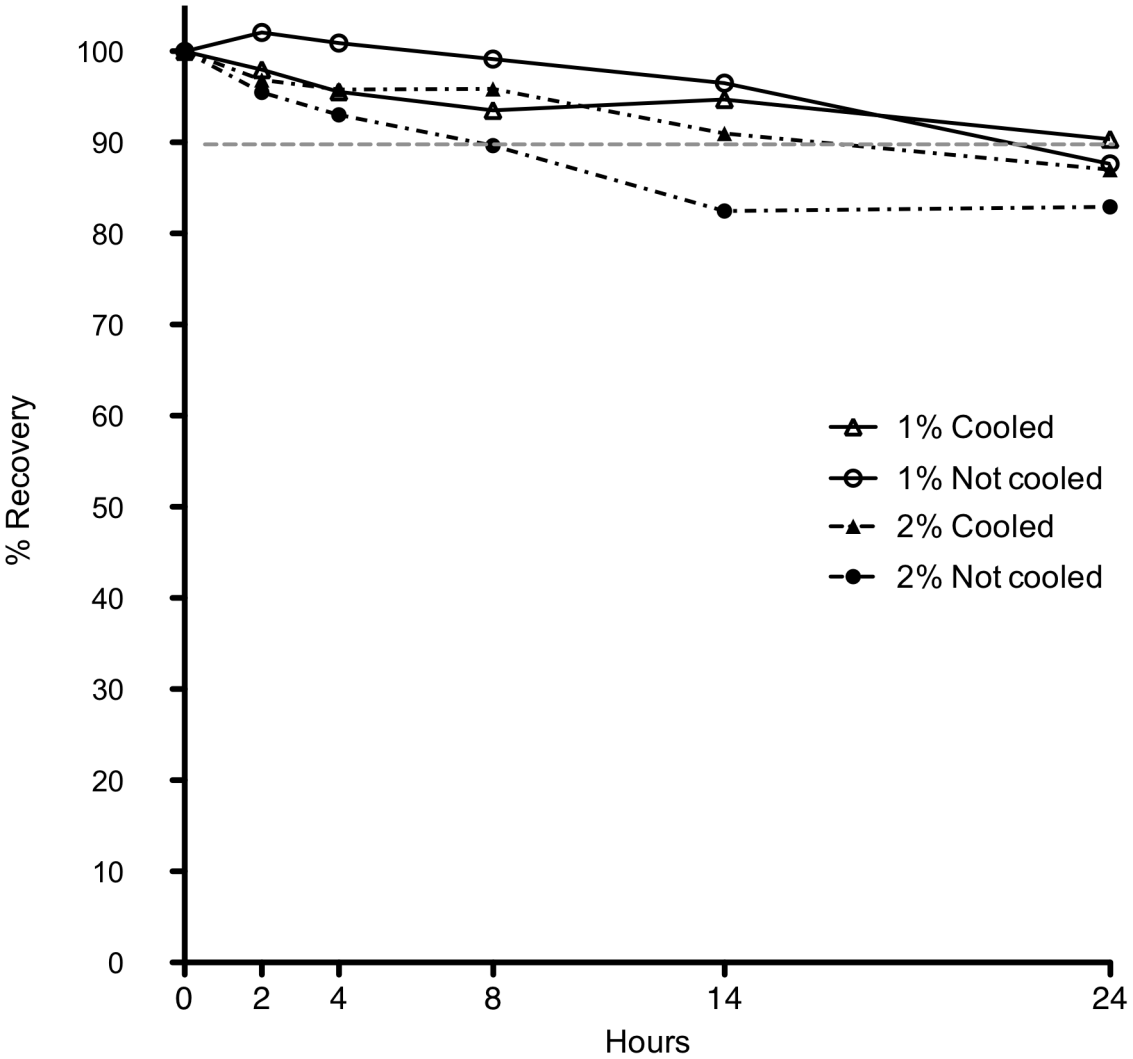
Meropenem degradation in elastomeric infusion devices at different conditions over a 24-hour period (data points are mean values).

A total of 32 infusion devices were prefilled. Three were excluded from further analysis because the initial concentrations were >25% expected. The 29 infusers included in the analysis were grouped as i) 1% w/v, room temperature (n = 6), ii) 2% w/v, room temperature (n = 7), iii) 1% w/v, cooled (n = 8), and iv) 2% w/v, cooled (n = 8) infusers. Meropenem recovery over the 24-hour period is shown (Figure 1). The mean 24-hour recovery, semi-quantitative temperature measurement and MDD% according to group are shown (Table 1). Cooled infusers with 1% w/v meropenem had a mean stability >90%. However, regardless of cooling, the 1% w/v infusers would deliver ≥95% of the MDD whilst only the 2% w/v infuser at room temperature delivered <90% of the MDD. Although the cooling measures increased the time that the temperature of the infuser was <20°C (12.3 versus 3.5 hours, *P*<0.0001), the time <20°C did not correlate with meropenem recovery at 24 hours (r^2^ = 0.04, *P* = 0.28). There were no statistically significant differences in median meropenem recovery at 24 hours between the infuser groups, regardless of concentration or cooling measures.

**Table 1:** Meropenem recovery, percentage of maximum deliverable dose and time less than 20°C for 24-hour elastomeric infusion device at different conditions (data are given as means [Standard Deviations; SD]).

Infusion concentration (w/v), cooled/uncooled	Time <20°C (hours)	Meropenem recovery at 24 hours (% [SD])	Maximum deliverable dose (% [SD])
1%, uncooled	3.68	87.6 (6.1)	96.4 (8.7)
2%, uncooled	3.34	82.9 (7.8)	87.2 (6.6)
1%, cooled	12.14	90.3 (6.5)	95.0 (5.7)
2%, cooled	12.39	87.0 (6.2)	92.9 (3.9)

**Figure 2:**
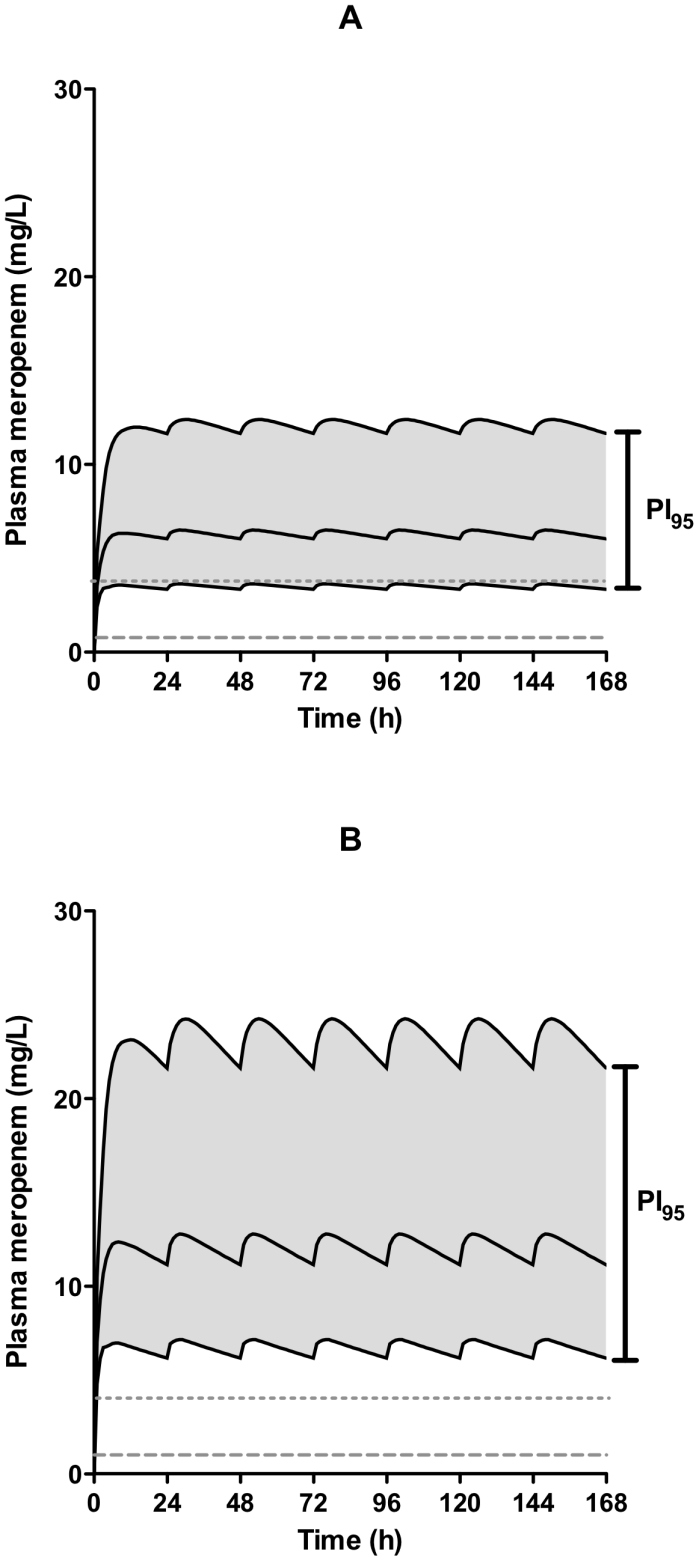
Median and 95% prediction intervals (PI_95_) of simulated plasma meropenem concentrations incorporating degradation as well as pharmacokinetic variability in 200 simulations of an average, 70 kg male patient receiving meropenem by continuous infusion in a cooled infuser containing 1% meropenem (figure 2A) and ‘room temperature’ infuser containing 2% meropenem (figure 2B). Susceptibility breakpoints for *Pseudomonas aeruginosa* (upper grey dashed line) and Enterobacteriaciae (lower grey dashed line) are also shown in each.

To illustrate of the potential effects of degradation on expected plasma meropenem concentrations in patients receiving continuous infusions, we incorporated population pharmacokinetic variability as well as our observed degradation to a simulated model of 70 kg male patients with normal renal function during a 7-day course of meropenem. Median, 2.5^th^ centile and 97.5^th^ centile plasma concentrations for 1% cooled and 2% room temperature infusers are shown in Figure 2A and 2B, respectively. In both simulations the vast majority had plasma concentrations above the breakpoints for *P. aeruginosa* and Enterobacteriaceae (4 mg/L and 1 mg/L, respectively) for 100% of the time.

## Discussion

With the progressive emergence of antimicrobial resistance in Gram-negative bacteria and the increased utilisation of OPAT for a variety of infections, there is considerable interest in re-examining the stability and safety of meropenem given by CI [16]. Our clinical study demonstrates a high rate of clinical efficacy (82% either cured or improved) with a low frequency of severe adverse events requiring cessation of therapy (10%). The present stability data demonstrate that infusers with meropenem at 1% under cooled conditions have a mean recovery at 24 hours that is equivalent to the traditional stability threshold of 90%. However, 1% infusers, regardless of cooling and cooled 2% infusers are likely to deliver close to 95% of the MDD of meropenem and therefore are likely to maintain effective plasma concentrations throughout each 24-hour period.

As observed in the present study, patients requiring prolonged courses of carbapenem antibiotics such as meropenem often have frequent co-morbidities, severe underlying chronic disease, a predominance of complex infections involving *P. aeruginosa* and resistant Enterobacteriaceae, and frequent concomitant administration of other antimicrobial medications. These factors may contribute to clinical failure and more frequent severe adverse events, particularly if the duration of therapy is prolonged [16].

Overall, the low frequency of haematologic toxicity (anaemia, thrombocytopaenia and neutropaenia), nephrotoxicity and/or hepatotoxicity was less than, or comparable to, that observed when other beta-lactams are used in the OPAT setting [16]. The toxicity profile of meropenem infusions in this study also compares favourably with that of vancomycin, an antibiotic commonly given by CI in our Western Australian OPAT centres, and which is associated with nephrotoxicity and neutropaenia occurring in 16% [17] and 2% [18] of patients, respectively. Our data also compare favourably with reported neutropaenia rates in patients receiving prolonged courses of piperacillin-tazobactam for bone infections [19].

To the best of our knowledge, no other study has examined 24 h meropenem stability in infusion devices at low concentrations under simulated OPAT conditions using practical cooling measures. The infuser devices in the present study were made up to 240 mL rather than the 100–125 mL used in other infuser devices [6], [7], thereby allowing the same dose to be infused but at more favourable lower concentrations. The mean recovery of 1% w/v meropenem carried in a cooled bag was 90% at 24 hours. In descending order, the mean 24-hour recoveries for infusers containing 1% w/v uncooled, 2% w/v cooled and 2% w/v room temperature meropenem were lower than the recommended 90% threshold (88%, 87% and 83%, respectively). The continuous temperature data show that, when using the ‘cooled’ bag with ice pucks changed every 8 hours, the temperature was <20°C for approximately half the time versus only ∼3 hours in the uncooled infusers. Although there was no statistically significant correlation between the time <20°C and mean meropenem recovery at 24-hours, meropenem recovery and MDD% were lower for the infusers that were kept at room temperature. In a comparable study, infusers containing 3% w/v meropenem were lodged between two ice-bricks and administered to patients with cystic fibrosis over 12 h. Continuous temperature measurements were not made during this study. After 12 h, the infusers remained cooled and were kept to assess meropenem recovery at 24 hours [7]. Using this method of infusion, plasma steady state concentrations remained well above the target concentrations required to treat most Gram-negative infections and the meropenem recoveries were all >90% after 12 h and in 3 of 4 infusers after 24 h [20].

Although the mean recovery of meropenem at 24 hours was ≤90% in our study, the time-dependent nature of meropenem degradation may mean that the total drug exposure is adequate to treat most serious infections. In the present study, we estimated the percentage of the MDD and found that, regardless of cooling measures, the patient would receive >95% of the planned dose with a 1% w/v continuous infusion and 93% and 87% for the 2% w/v infusers in cooled and room temperature bags, respectively.

The illustrative simulations also demonstrate that adequate pharmacodynamics targets are likely to be attained in most patients, regardless of the infecting organism. These simulations were based on inputs that accounted for degradation during the infusion and incorporated published population pharmacokinetic variability for meropenem. They demonstrate that the simulated median meropenem concentrations of 200 average 70 kg, male adult patients receiving continuous CIs are above accepted breakpoints [15] for *P. aeruginosa* and Enterobacteriaceae 100% of the time, regardless of the cooling measures undertaken. The 2.5^th^ centile of these simulations also demonstrates that nearly all patients receiving 2%, uncooled meropenem infusors are also above both breakpoints for 100% of the time, but indicate that a small minority of patients receiving 1% infusers may not achieve concentrations above 4 mg/L for 100% of the time.

The other major factor in determining a safe stability threshold for meropenem is the potential toxicity of degradation products. A thermal degradation product (DP1) of meropenem in aqueous solution following a 36 hour exposure to 45°C has been identified as 4-methyl-3-(1H-pyrrol-3-ylsulfanyl)-5H-pyrrole-2-carboxylic acid (MW = 227.1). Few clinical data exist on the possible toxicity profile of DP1. In the only relevant paper in the literature, a degraded solution of meropenem had a limited cytopathic effect on *in vitro* cultured peripheral blood mononuclear cells at 24 h at concentrations 0.5–2 mg/mL, but some effect after incubation for 48 and 72 h [21].

Our study had some limitations. The clinical part was retrospective and therefore the clinical data may not have been collected in a standardised manner. However, we believe that most important possible adverse events were captured as the OPAT clinicians reviewed all patients at least weekly. In addition, laboratory monitoring was performed at least weekly for all OPAT patients in a standardised manner. Although we did not include a control comparator group, the substantial differences in co-morbidities, microbiological susceptibility of infecting organisms and illness severity (defined by in-patient status), precluded robust matching of our case patients to possible controls such as OPAT patients receiving piperacillin/tazobactam by continuous infusion or to inpatients receiving intermittent infusions of meropenem. As this was a retrospective study and our laboratory does not routinely perform meropenem MICs for Gram-negative organisms, we were unable to adjust our analyses of clinical outcome according to the infecting pathogen's MIC. A final limitation was that the simulations did not account for potential variability in renal function. In the real world, as demonstrated in the present case series, renal function may be impaired in patients with multiple co-morbidities and treated with concomitant potentially nephrotoxic medications, resulting in decreased clearance of meropenem. Conversely, very sick patients may have augmented renal clearance and increased drug distribution that might result in inadequate drug exposure. A formal analysis of such effects was beyond the scope of the present study, not least because these very unwell patients with significant pharmacokinetic perturbations would not usually be suitable for OPAT.

In summary, the present study demonstrates that infusions with meropenem at 1% w/v under cooled conditions have a mean recovery of 90% at 24 hours, a value equivalent to the stability threshold of 90%. However, 1% w/v solutions regardless of cooling and cooled 2% w/v solutions deliver close to 95% of the MDD. Simulated data that incorporates population pharmacokinetic variability as well as the degradation observed also demonstrate that for the majority of patients, meropenem concentrations will be maintained above the breakpoints for Enterobacteriaceae and *Pseudomonas aeruginosa* for 100% of the time.

Although these results accord with the favourable clinical experience at our institutions and are reassuring because adequate drug concentrations are likely to be attained, further studies are required to confirm the safety and efficacy of meropenem CIs. Ideally, future studies should be prospective with pre-defined assessments of toxicity and efficacy outcomes. Meropenem and possible toxic degradation products should also be measured in patient plasma samples.
